# Case Report: Auditory Hallucination Induced by Amitriptyline for the Treatment of Atypical Odontalgia

**DOI:** 10.3389/fpsyt.2022.863485

**Published:** 2022-05-02

**Authors:** Motoko Watanabe, Tetsuo Nakabayashi, Gayatri Nayanar, Chihiro Takao, Chizuko Maeda, Trang Thi Huyen Tu, Haruhiko Motomura, Akira Toyofuku

**Affiliations:** ^1^Department of Psychosomatic Dentistry, Graduate School of Medical and Dental Sciences, Tokyo Medical and Dental University, Tokyo, Japan; ^2^Department of Psychiatry and Behavioral Sciences, Graduate School of Medical and Dental Sciences, Tokyo Medical and Dental University, Tokyo, Japan; ^3^Department of Basic Dental Sciences, Faculty of Odonto-Stomatology, University of Medicine and Pharmacy at Ho Chi Minh City, Ho Chi Minh City, Vietnam

**Keywords:** auditory hallucination, amitriptyline, atypical odontalgia, chronic pain, case report, adverse events, antidepressants

## Abstract

Auditory hallucination is usually associated with psychiatric diseases and organic brain illness. It was rarely found as adverse events of antidepressants. Amitriptyline is considered as one of the first line medications for the psychopharmacotherapy of chronic pain including atypical odontalgia (AO) which shows chronic tooth pain without corresponding abnormalities. Anticholinergic adverse events induced by amitriptyline are usually bearable and not critical since the prescription dose is very low for the patients with AO. This is a first case report about the AO patients who showed auditory hallucination by the low dose of amitriptyline. A 43-years-old female, housewife, complained chronic toothache following dental procedures and was diagnosed as AO. Amitriptyline was initially prescribed 25 mg and gradually increased up to 60 mg with the improvement of AO symptoms in 7 months. Although the temporary recurrence was observed following to the retreatment of prosthodontic dental procedures, it improved in a few weeks. Therefore, the dose of amitriptyline was decreased, and the continuation dose was set 30 mg. In 24 months, the AO symptoms were very much improved; however, she reported that she had been heard the voices at midnight for a year. The voices were neighborhoods' and talking about the noise troubles she had claimed before. She had not realized that the voices were auditory hallucination since they were heard only at midnight infrequent and not bothering her daily life. At the time she reported auditory hallucination, she worried whether organic brain diseases are hiding because the frequency of voices was increased and sometimes occurred in daytime. The adverse event of amitriptyline was suspected since she had never had psychotic symptoms before. Amitriptyline was decreased and continued with the dose of 25 mg. Magnetic resonance imaging and psychiatric consultation revealed no abnormality of brain and in psychiatric aspects. After final prosthodontic treatment, the amitriptyline was discontinued in 30 months. Two months after the discontinuation, auditory hallucination was almost disappeared with no recurrence of AO. The present case report suggests that amitriptyline has possibility to induce auditory hallucination even in conventional dose throughout the treatment of chronic pain including AO.

## Introduction

Auditory hallucination is generally associated with psychotic disease such as schizophrenia, and depression, dementia, and other organic brain lesions; moreover, sometimes it is induced by antidepressants (1–4). Amitriptyline, a tricyclic antidepressant has been used in the treatment not only for major depression but also chronic pain. Its side effects are well known of dry mouth, constipation, drowsiness, cardiovascular effects and orthostatic hypotension. In the psychopharmacotherapy of chronic pain with amitriptyline, since the prescription dose is quite low, the most adverse events were related to anticholinergic affect but generally tolerable. Hence, we reported a rare case of auditory hallucination induced by the low dose of amitriptyline in the treatment of atypical odontalgia (AO), a persist idiopathic pain in tooth and other orofacial area (5).

## Case Description

A 43 years-old female, housewife, visited our department with her chief complaint of chronic toothache in bilateral upper molars. In May X-1 years, she had prosthodontic treatments following root canal treatments. In September X-1 years, she felted continuous toothache which was exacerbated by chewing, cold and hot food intake. She visited several dental clinics and otolaryngology clinics; however, there was no corresponding abnormalities. Then she was referred to a pain clinic in the university hospital in October X-1 years. The treatment with amitriptyline 10 mg and pregabalin 100 mg did not improve her pain symptoms though and was discontinued after 6 months, because of her moving to Tokyo. She visited several dental clinics again and was referred to our department in May X years.

**Figure 1:**
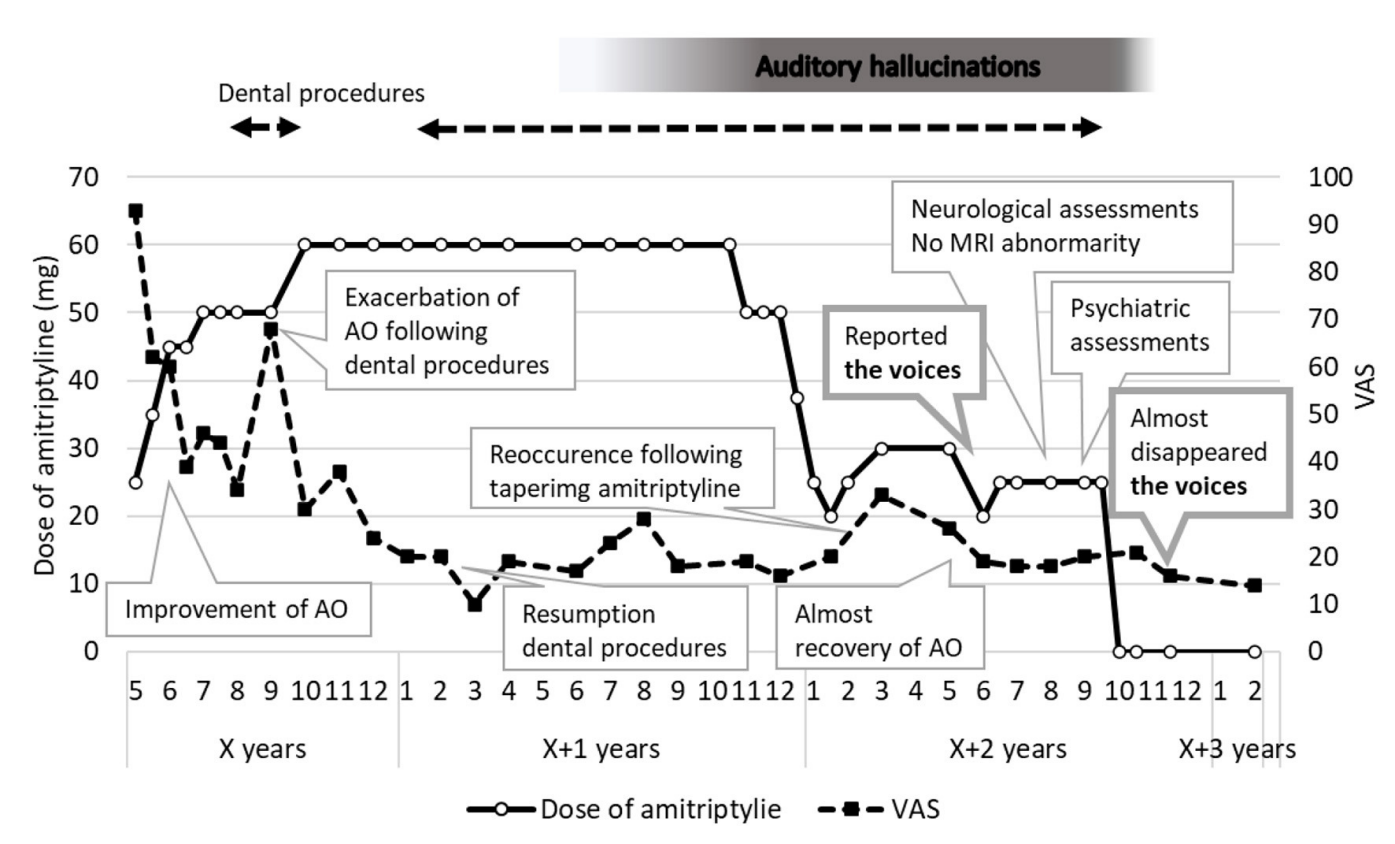
The timeline of treatment prognosis.

There were no abnormalities such as caries, clacks, and periapical lesions aligned to her complaints. We diagnosed her condition as AO and prescribed amitriptyline. Since the dose of 10 mg was not effective before and had not been increased, 25 mg was set initially and gradually increased to 45 mg (Figure 1). AO symptoms were improved in June X years. Adverse effects such as drowsiness, dry mouth and constipation were observed but well controlled by herself. Following dental procedures. AO symptoms had exacerbated. Therefore, the dose was increased up to 60 mg since October X years and significant improvement of her symptoms was observed in January X+1 years. The prosthodontic retreatments of some teeth were restarted in February X+1 years. During dental procedures, sometimes there were temporary recurrences, but they all quickly improved in a few weeks. In November X+1 years, the tapering was started but in March X+2 years, a recurrence was observed with 20 mg of amitriptyline prescription. Since continuing 25 mg did not improved symptoms enough, 30 mg was set as a maintenance dose in April X+2 years. In May X+2 years, the symptoms were almost disappeared; however, she told us she had heard auditory hallucination for a year since June X+1 years.

She had not reported the voices she had heard for a year because she had not realized that the voices were auditory hallucinations. The voices sounded like neighbors' and clear enough to understand what they were talking about. She reported that her neighbors were always arguing about the noise troubles she had claimed to them before. The voices were appeared infrequently, only when she waked up at mid-night or around 2–3 a.m. There was no significant change in her life including over-the-counter drugs, psychosocial stresses, or behavioral changes. Six months after, when hearing the same voices at outside in daytime, she thus realized that they might be auditory hallucinations. After a few days, the daytime voices disappeared, and the voices became to be found only at nighttime again. Sometimes, at midnight, she even waked her husband up to ask if he can hear the same voices. Auditory hallucination did not threaten her daily life much, instead it made her anxious and wonder if there are any hidden organic diseases like brain tumor.

At first, since the patients had no psychiatric history and did not show any other psychogenic symptoms, we consider the adverse event induced by amitriptyline. Amitriptyline was then decreased to 20 mg; however, AO symptoms exacerbated in a few days and the dose of 25 mg was continuously prescribed in July X+2 years. After our referral to neurologists in August X+2 years, the magnetic resonance images (MRI) revealed neither cerebrovascular disease nor brain tumor. She was then referred to psychiatrists where she was suggested to stop taking amitriptyline to assess the adverse effects. Considering that AO symptoms did not get worse during the final prosthodontic procedure, the medication was discontinued in September X+2 years.

The neighbors' voices as auditory hallucinations were found since June X+1 years and gradually disappeared after discontinuation of amitriptyline.

## Diagnostic Assessment

The auditory hallucinations were heard only in the early morning when she woke up. It might be in hypnopompic; however, she was wide awake and remember the voices clearly. To identify the noise source, she turned off some home appliances such as fans, air conditioners which usually make small sounds in daily life and even wake her husband up to ask if he can hear the voices. The neurological assessment revealed no considerable possibilities of migraine, undiagnosed central nervous system lesions or latent epilepsy. Since no organic brain disease was observed by MRI and no psychotic disease (e.g., schizophrenia, abuse of alcohol or drugs, or symptoms such as delusional idea) was found, we diagnosed her symptoms as auditory hallucination induced by amitriptyline.

## Follow-Up and Outcomes

Amitriptyline was discontinued in September X+2 years. In the next 2 weeks, auditory hallucinations maintained but less frequent and gradually became vague in the third week. In November X+2 years, the auditory hallucination almost disappeared. She reported that she sometimes sensitive to sounds in her daily life but no longer could hear clear voices. Psychiatric follow-up has ended. The voices completely disappeared, and recurrence of AO was not observed in February X+3 years. The follow-up of our department has also ended.

## Discussion

Amitriptyline is suggested as the first line medication of chronic pain in the orofacial region, including AO (5). Among several adverse events of amitriptyline that are particularly related to anticholinergic symptoms, auditory hallucination is seldom observed. This report presents an auditory hallucination case (besides constipation and dry mouth) with conventional dose of amitriptyline in the patient with AO.

Generally, when auditory hallucinations are observed, at first the possibility of hidden diseases should be considered, such as schizophrenia, dementia, brain tumor and so on. Secondly, the practitioners should be prudent to assess adverse events of medication with considering the cases who do not realize the symptoms are adverse events as well the present case. In literature, there are reported cases in which auditory hallucinations were induced by selective serotonin reuptake inhibitors (SSRI) such as citalopram (3), paroxetine (1), and trazodone (2). Since all of them have interactions with serotonin, the possible involvement of serotoninergic systems in the present of auditory hallucinations was evidenced. At the same time, imipramine also showed musical hallucination, which is one type of auditory hallucinations (4). For the tricyclic antidepressants (TCA) (including imipramine and amitriptyline), auditory hallucinations might be related to anticholinergic effects; moreover, they also might be affected with the serotoninergic system of TCA as previous case reports of SSRI. Hence, not only anticholinergic affects but also serotoninergic neurotransmitters might be involved in auditory hallucinations induced by TCA.

In addition, the auditory hallucination in hypnagogic and hypnagogic state should be concerned. It is typically unclear, not recurrent, the voice of known people, likely direct talk and not dialogical (6). In this case, the voices she had heard were like neighbors' and not direct to the patient; however, that were clear and recurrent. From the point of that she was awake when she had heard the voices as well, the voices in this case were differ from the auditory hallucinations in the hypnagogic and hypnopompic.

Moreover, after amitriptyline discontinuation, she reported that she still heard the same voices occasionally in her daily life but immediately recognized as just the daily sounds. Since it takes 4 months for her auditory hallucinations to completely disappear, we suggested the brain hypersensitivity might be induced by amitriptyline. She was taking 60 mg of amitriptyline for a year, therefore the long-lasting effect of brain hypersensitivity probably remained even after discontinuation of amitriptyline.

Furthermore, in the previous reported cases, medications were prescribed as the treatment of psychiatric diseases, such as major depression, obsessive-compulsive disorders, dysthymia, depressive states. In our present case, amitriptyline was for AO management. Generally, since the dose of amitriptyline that usually prescribed for the treatment of chronic pain is quite low, the severe adverse events are seldom observed (5). In this case, although she did not report auditory hallucinations for a year, she had heard the voices sometimes during the intake of amitriptyline 60 mg. In addition to dry mouth and constipation which were within her tolerance, auditory hallucination was clearly found. In cases who present no psychiatric history, the serum level of medications tends to be high so adverse events would be easily observed and remain, even in the low dose. This is the first case report of auditory hallucinations induced by antidepressant monotherapy for AO. Furthermore, the recurrence of chronic pain symptoms should be cautious during tapering of medication. In this case, AO symptoms reoccurred when conducting dental procedure and when decreasing prescription dose. Because of the dilemma between the efficacy of amitriptyline and adverse events, it usually takes time to discontinue taking amitriptyline. Further studies about therapeutic dose of amitriptyline for the treatment of AO are needed.

While the auditory hallucinations in this case were inappropriate for that in hypnagogic and hypnopompic or that induced by psychotic diseases, they were also atypical as drug-induced auditory hallucinations in the point of limited appearance. However, since the voices disappeared following the discontinuation of amitriptyline, it is reasonable to suggest that the symptoms were induced by amitriptyline.

The present case suggests that amitriptyline has possibility to induce auditory hallucination, even in conventional dose in the patients with AO.

## Data Availability Statement

The original contributions presented in the study are included in the article, further inquiries can be directed to the corresponding author.

## Ethics Statement

The studies involving human participants were reviewed and approved by the Ethical Committee of Tokyo Medical and Dental University Dental Hospital. The patients/participants provided their written informed consent to participate in this study. Written informed consent was obtained from the individual(s) for the publication of any potentially identifiable images or data included in this article.

## Author Contributions

MW was involved in the treatment of the presented cases, writing of the first draft, and editing the manuscript. TN, GN, CT, and CM were involved in patient treatment. TT and HM reviewed and edited the manuscript. AT treated patients and was a major contributor in writing the manuscript. All authors have read and approved the manuscript.

## Funding

This research was funded by KAKENHI from the Japanese Society for the Promotion of Science (JSPS), Grant Number 19K10328 to AT and Grant Number 19K19211 to MW. The funder had no role in study design, data collecting, data analysis, decision of publishing, and preparation of the manuscript.

## Conflict of Interest

The authors declare that the research was conducted in the absence of any commercial or financial relationships that could be construed as a potential conflict of interest.

## Publisher's Note

All claims expressed in this article are solely those of the authors and do not necessarily represent those of their affiliated organizations, or those of the publisher, the editors and the reviewers. Any product that may be evaluated in this article, or claim that may be made by its manufacturer, is not guaranteed or endorsed by the publisher.
